# Does remote match reality? Comparing the effectiveness of a self‐help app for panic disorder and agoraphobia to face‐to‐face CBT

**DOI:** 10.1111/papt.70012

**Published:** 2025-09-01

**Authors:** Justine Spies, Thomas Lang, Alexander L. Gerlach, Tilo Kircher, Alfons Hamm, Georg W. Alpers, Thomas Fydrich, Volker Arolt, Jürgen Deckert, Andreas Ströhle, Hans‐Ulrich Wittchen, Sylvia Helbig‐Lang

**Affiliations:** ^1^ Universität Hamburg Hamburg Germany; ^2^ Christoph‐Dornier‐Stiftung Institut Bremen Bremen Germany; ^3^ Constructor University Bremen gGmbH Bremen Germany; ^4^ Universität Zu Köln Klinische Psychologie Und Psychotherapie Köln Germany; ^5^ Universitätsklinik für Psychiatrie und Psychotherapie Marburg Germany; ^6^ Universität Greifswald Greifswald Germany; ^7^ University of Mannheim, School of Social Sciences and Otto Selz Institute Mannheim Germany; ^8^ Humboldt‐Universität zu Berlin Berlin Germany; ^9^ University Hospital Münster Münster Germany; ^10^ Universitätsklinikum Würzburg Würzburg Germany; ^11^ Charité Universitätsmedizin Berlin Berlin Germany; ^12^ IAP‐AVM‐Dresden GmbH Dresden Germany; ^13^ PTA Hamburg Psychotherapieausbildung an der Universität Hamburg Hamburg Germany

**Keywords:** agoraphobia, cognitive behavioural therapy, panic disorder, post‐hoc‐comparison, smartphone‐based

## Abstract

**Background:**

Exposure‐based CBT is highly effective in treating patients with panic disorder and agoraphobia; however, access to such treatments is often limited. Smartphone‐based self‐management apps offer a promising low‐threshold treatment alternative to face‐to‐face therapy. Although such health apps have shown to be effective in reducing anxiety symptoms, comparisons to active treatments are still scarce. Therefore, this study compared the effectiveness of a self‐help app to an established face‐to‐face CBT intervention for panic and agoraphobia.

**Method:**

The present study conducts a post hoc comparison of two independent RCTs examining participants with panic disorder and/or agoraphobia. Interventions in both studies were based on the same CBT manual. Study 1 (*n* = 138) included face‐to‐face CBT; Study 2 addressed the effects of a digital self‐help intervention (*n* = 57). Main outcomes comprised symptoms of both panic disorder and agoraphobia, depressive symptoms and agoraphobic avoidance. Data were analysed using linear mixed models in intent‐to‐treat and completer data sets.

**Results:**

Linear mixed models showed that face‐to‐face treatment was superior to app treatment in reducing panic and agoraphobic symptoms (*R* ^2^ = 0.32), depressive symptoms (*R* ^2^ = 0.24) and agoraphobic avoidance (*R* ^2^ = 0.12 and 0.15). Dropout rates did not differ significantly, and both interventions demonstrated high levels of adherence.

**Discussion:**

Although a smartphone‐based CBT intervention was effective in reducing symptoms of panic and agoraphobia, its efficacy was significantly below the effects of the same intervention delivered in face‐to‐face format. Thus, digital interventions might be most suitable within a stepped‐care approach or to bridge waiting times for psychotherapy.

## INTRODUCTION

Cognitive behavioural therapy (CBT) is currently considered best practice treatment for panic disorder and/or agoraphobia (PD/A, Bandelow et al., 2021; NICE, 2020). However, only a minority of individuals receive appropriate and sufficient care; for instance, because of long waiting times for psychotherapy (Neutens, 2015; Villatoro et al., 2022). Digital mental health interventions (DMHIs) have gained popularity in the last few years as a low‐threshold treatment alternative to face‐to‐face psychotherapy. DMHIs can be implemented as computer programmes, browser‐based web programmes or as smartphone‐based apps (Maaß et al., 2022), and are, thus, easily accessible, especially in more remote areas (Weightman, 2020). Moreover, they are cost‐effective and straightforward to use (e.g. Andersson et al., 2014; de Vries et al., 2021; Heron & Smyth, 2010). Research has already shown that DMHIs are highly effective in reducing PD/A symptoms (e.g. Andersson et al., 2014; Andrews et al., 2010; Pauley et al., 2023; Stech et al., 2020). A recent meta‐analysis by Stech et al. (2020) found that DMHIs outperformed waitlist control groups and information controls with effect sizes of Hedges' *g* = 1.22 for panic disorder and *g* = 0.91 for agoraphobia. This is in line with findings by Pauley et al. (2023) who reported an effect size of *g* = 1.08 for DMHIs for PD/A in comparison with inactive control groups. One meta‐analysis and review by Domhardt et al. (2020) also compared DMHIs to active control groups (face‐to‐face (F2F) CBT and applied relaxation) and did not find significant differences in panic and agoraphobic symptom reductions. A benchmark study by Strauss et al. (2022) also found therapist‐guided DMHI within‐group effects (0.88–1.7) similar to effects in F2F CBT, further supporting DMHI effectiveness. Most recently, a systematic review and meta‐analysis by Hedman‐Lagerlöf et al. (2023) compared therapist‐supported internet‐based CBT to F2F CBT and found similar effectiveness for a wide range of mental and somatic disorders.

However, these studies examined DMHIs with therapist guidance. As therapeutic capacities are costly and often sparse, the question remains whether DMHIs as stand‐alone treatments are a potential alternative to standard treatment. A recent meta‐analysis by Seegan et al. (2023) suggested that smartphone‐based stand‐alone DMHIs (CBT‐, Mindfulness‐ or Acceptance and Commitment‐based) had a small effect on reducing anxiety severity compared to control conditions (*g* = 0.31). As this meta‐analysis included both clinical and non‐clinical samples and did not distinguish between different anxiety disorders, it remains unclear whether these effects might generalize to specific clinical samples. Indeed, subgroup analyses suggested that treatment effects might be stronger in clinical samples. Descriptive results also indicated that CBT interventions may offer advantages over other treatment approaches. The authors also pointed out that only limited information was available regarding user engagement with the DMHIs, raising the question of whether results might be attributed to a lack of engagement with the app's content.

Taken together, there is evidence that DMHI in general is effective in treating panic and anxiety disorders. DMHIs as stand‐alone treatments have yielded some support in reducing anxiety symptoms; however, due to the limited number of studies on specific populations and the lack of studies using state of the art treatment as a comparison condition, more research is warranted to conclude which effects can be expected for whom when implementing a DMHI.

In order to address this question, we compared the effects of a smartphone‐based self‐management DMHI to a F2F CBT treatment study (Gloster et al., 2009, 2011). Notably, both studies examined clinical populations diagnosed with panic disorder and/or agoraphobia, and the interventions in both studies relied on the same treatment manual that had been shown to be effective in treating PD/A (Gloster et al., 2011). Given the established impact of agoraphobic avoidance (e.g. Porter & Chambless, 2015) and depressive symptoms (e.g. Bruce et al., 2005) on treatment effects in PD/A, we examined these variables as secondary outcome measures. Finally, we were interested in adherence rates in both conditions as an indicator of acceptance and tolerability.

## METHOD

Analyses were based on post hoc analyses of data from the active treatment groups of two independent RCTs. Studies providing data are described below.

### F2F study

#### Study design

The sample for the F2F comparison condition was drawn from a multicentre trial where participants were randomized to one of three conditions (CBT with therapist‐guided exposure vs. CBT with self‐management exposure vs. waitlist control group). For the present analyses, participants assigned to the self‐management exposure group were examined. The study was approved by the Ethics Committee of the Medical Faculty of the Technical University of Dresden (EK 164082006) and was funded by the German Federal Ministry of Education and Research (BMBF, project number: 01GV0615). For study details, see Gloster et al., 2009, 2011).

#### Participants

In order to be included in the study, participants had to (a) meet the DSM‐IV‐TR diagnostic criteria of a panic disorder and/or agoraphobia, (b) have a clinical interview score of ≥18 on the structured interview guide for the Hamilton anxiety scale (SIGH‐A, Shear et al., 2001), (c) a score of ≥4 on the clinical global impressions scale, (d) be 18–65 years of age and (e) be able to regularly attend treatment sessions. Participants were excluded if they had (a) a comorbid psychotic or bipolar I disorder, (b) a current alcohol dependence/current abuse or dependence for benzodiazepine and other psychoactive substances, (c) were suicidal, (d) had a borderline personality disorder, (e) were currently undergoing psychotherapeutic or psychopharmacological treatment for any mental disorder and (g) had physician‐verified contraindications of exposure‐based CBT such as cardiovascular, renal or neurological diseases (see Appendix B for a study flow chart).

#### Treatment

Both active treatment groups in the F2F study received outpatient CBT based on the treatment manual for panic disorder and agoraphobia (Lang et al., 2012). The main difference between the active groups was the implementation of the in vivo exposure component: Participants in one group underwent therapist‐guided in vivo exposure; the other group received instructions but completed the exercises independently, as part of a self‐managed approach. For the present comparison, only participants in the unaccompanied group were considered as this treatment procedure matches more closely to a self‐management DMHI.

The underlying treatment manual consisted of 12 individual CBT sessions, which were conducted over approximately 8 weeks. Sessions 1–3 were focused on psychoeducation, sessions 4–5 introduced interoceptive exposure. Sessions 6–8 consisted of planning and conducting three standardized in vivo exposure exercises, Session 9 addressed progress and changes in anticipatory anxiety, and sessions 10–12 were focused on planning and discussing individual exposure exercises. Afterwards, two booster sessions were offered to discuss individual exposure exercises and for relapse prevention. Homework was assigned in each session and included self‐monitoring of panic attacks and agoraphobic avoidance, reflecting on psychoeducational information, symptom provocation exercises and repeating in vivo exposure exercises (for details see Appendix D).

#### Assessments

Five assessments were scheduled including an intake assessment (T1) prior to enrollment in the study, the baseline assessment before treatment start (T2), an intermediate assessment (after the fourth session, T3), a post assessment (T4, at the end of treatment) and a follow‐up assessment (6 months after the end of treatment, T5). Outcome measures were evaluated at each assessment point. For the present analyses, data from baseline and post‐assessment were used.

#### Measures

#### Diagnostic Status

Diagnostic status was assessed by means of the Composite International Diagnostic Interview (CIDI, Essau & Wittchen, 1993, Wittchen & Pfister, 1997). The CIDI is a standardized computer‐administered interview that assesses DSM‐IV mental disorders.

#### Severity of Panic and Agoraphobic Symptoms

The primary analyses by means of LMM to compare the app and the F2F treatments were based on the self‐assessed severity of panic and agoraphobic symptoms by means of the German version of the Panic and Agoraphobia Scale (PAS, Bandelow, 2016) which has proven to be useful in measuring treatment efficacy (Bandelow et al., 1998). The PAS consists of the five subscales: panic attacks, agoraphobic avoidance, anticipatory anxiety, impairment in social relationships and work and assumptions of somatic disease.

#### Depressive Symptoms

The German version of the revised Beck Depression Inventory (BDI‐II, Hautzinger et al., 2006) was used to evaluate whether the two groups differed in depressive symptoms at baseline. Comorbid depressive symptoms in anxiety patients have been shown to be related to slower recovery and a higher risk of recurrent anxiety symptoms (e.g. Bruce et al., 2005). Thus, significant group differences would distort analyses of treatment effects. The self‐report questionnaire BDI‐II consists of 21 items where one is asked to evaluate the severity of different depression symptoms. Its' internal consistency is good (e.g. Jackson‐Koku, 2016).

#### Agoraphobic Avoidance

The Mobility Inventory (Chambless et al., 1985) was used to compare the two groups in terms of their levels of agoraphobic avoidance at baseline, as agoraphobic avoidance has been found to be a predictor of less improvement (Porter & Chambless, 2015). The self‐report questionnaire with scores ranging from 1 to 5 assesses agoraphobic behaviour and frequency of panic attacks in 26 situations whilst being alone or accompanied. The internal reliability is good (Chambless et al., 1985).

#### Adherence

The completion of homework served as an indicator of treatment adherence. Homework compliance was evaluated directly after each session by the therapist and patients using specific protocols. Patients rated their homework compliance on 7‐point Likert scales ranging from 0 (not at all) to 6 (completely done). Therapists were requested to provide a thorough evaluation of the quantity and quality of homework completion using categorical ratings. The quantity of homework completion was rated as 0 (not at all), 1 (less than assigned), 2 (as assigned) or 3 (more than assigned). Quality was rated as 0 (unsatisfactory), 1 (satisfactory), 2 (good) or 3 (excellent). The required amount of homework was outlined in the treatment manual. Its quality was assessed based on the appropriate application of the treatment rationale.

### App study

#### Study design

Data were drawn from a prospective multicentred two‐armed RCT that examined the efficacy of the smartphone‐based self‐management app ‘Mindable’ (Spies et al., 2024). In this study, participants were randomly assigned either to an intervention group that received the DMHI or a waitlist control group. Ethical approval was granted by the ethical committee of the German Society for Psychology (reference number: LangThomas2020‐12‐14VA) and the study was preregistered with the Clinical Trial Registration (registration number: DRKS00029090) and conducted in agreement with CONSORT guidelines. Detailed information can be found in (Spies et al., 2024).

#### Participants

Inclusion and exclusion criteria were similar to the F2F study, but did not include requirements for symptom severity. Participants (Spies et al., 2024) had to meet the DSM‐5 diagnostic criteria of panic disorder and/or agoraphobia, and had to be at least 18 years of age. Participants were excluded if they a) were currently undergoing psychotherapy, (b) had no smartphone, (c) had a change in medication in the last 2 months or were taking benzodiazepines, (d) suffered from a comorbid substance use or psychotic disorder, (e) had comorbid chronic respiratory or cardiovascular diseases, (f) were suicidal, (g) did not have sufficient German language abilities or (h) were unable to read or write (see Appendix A for a study flow chart).

#### Treatment

Participants in the active study group were introduced to the ‘Mindable’ app. App development was based on the treatment manual used in the F2F study and comprised similar contents and methods (see Appendix D for a comparison of treatment contents). Within a brief introduction, participants were advised to complete the modules in the same sequence as used in the F2F study; however, they were free to navigate the app in a different order if they preferred.

The app included the module ‘psychoeducation’ comprising nine lessons on the aetiology and maintenance of panic disorder and agoraphobia. Participants were advised to start with this module. The second module ‘symptom provocation’ provided information about interoceptive exposure and offered instructions for exercises such as spinning or hyperventilating. A third module ‘in vivo exposure’ informed about aims and procedures of exposure in vivo and provided suggestions and protocols for specific exposure exercises. In contrast to the F2F study, no standardized exercises were suggested; participants could choose exercises in accordance with their individual fear hierarchy. The app also provided reminders for planned exercises and charts depicting anxiety curves within and across exercises, and featured a daily diary and a weekly check‐up, allowing users to record their anticipatory anxiety, panic attacks, avoidance behaviours and current symptom state similar to the documentation used in the F2F study.

#### Assessments

In the baseline‐assessment (T1) participants gave their informed consent, structured diagnostic interviews were conducted, and all outcome measures were assessed. After 4 weeks, participants were invited again to complete the questionnaires online in the between‐assessment (T2). Eight weeks after the baseline‐assessment, the structured diagnostic interview as well as all outcome measures were repeated at the post‐assessment (T3).

#### Measures

#### Diagnostic Status

In the app group, the Diagnostic Interview for Mental Disorders—Open Access (DIPS‐OA, Margraf et al., 2017) was used to evaluate whether participants met inclusion and any exclusion criteria. It assesses mental disorders according to the DSM‐5. The interviews were conducted at baseline in the study centres or via certified video software (RED Medical, 2014).

#### Symptom Severity and Depressive Symptoms

Outcome measures were identical to those used in the F2F study and included the PAS, the MI and the BDI‐revised (for details, see Section 2.1.5).

#### Adherence

An adherence score was defined a priori based on clinical judgements and was employed as an indicator of treatment integrity. To calculate the adherence score, recommended usage criteria were defined for each app module. The adherence score reached 100% when the recommendations were fully met or exceeded. Categories for lower adherence scores were established for cases where the recommended levels were not met. Three adherence scores were calculated for (1) the module ‘psychoeducation’, (2) the ‘check‐up’ and ‘diary’ sections and (3) the number of exercises in the modules ‘symptom provocation’ and ‘exposure’. For example, a 100% adherence score was achieved if participants (1) completed nine out of nine psychoeducation lessons, (2) had at least eight entries in the ‘check‐up’ and ‘diary’ sections and (3) had conducted at least five exercises. Based on these three scores, an average and overall adherence score was calculated. An overall adherence score of 75% or above was considered indicative of adherence.

### Statistical analyses

Within‐group effects were evaluated by means of pre‐post‐comparisons and the calculation of Cohen's *d* (*d* < 0.5 = small, 0.5 < *d* < 0.8 = middle and *d* > 0.8 = strong effect, Cohen, 1988). In order to compare the two intervention groups, linear mixed models (LMM) were calculated. As missing values were expected to be missing at random, missing values were handled with multiple imputation. Based on the proportion of missing data 20 imputations per missing value were generated (in accordance with Graham et al., 2007), and the results were pooled in accordance with Rubin's rules (Rubin, 1987). All variables relevant to the LMM were used as predictors. As the potential scale reduction factor ($\hat{R}$) scores were all <1.05 and the visual inspection of the trace plots indicated stable variation around a constant equilibrium, convergence was deemed achieved. The dependent variables in the LMM were the scores of the PAS in general, depressive symptoms measured by means of the BDI‐II and agoraphobic avoidance measured by means of the MI. For the purpose of controlling for individual differences in participants, the variable ‘participant’ was used as a random effect. The variables ‘group’ (app vs. face‐to‐face), ‘time’ (baseline vs. post), the group‐by‐time interaction as well as ‘employment status’ (unemployed yes/no) and ‘comorbid depressive disorder’ (yes/no) were used as fixed effects, as the latter two were found to differ significantly between the app and F2F group. The calculated models were compared to a ‘null model’, which lacked the interaction term. The difference in variance explained by each of the two models (*R* ^2^) was calculated in order to determine the variance explained by the interaction between ‘time’ and ‘group’.

Response was determined by calculating the Reliable Change Index (RCI, Christensen & Mendoza, 1986; Jacobson et al., 1984). It was calculated by means of the PAS' norm sample (*r*_tt_ = 0.78). Hence, reliable change was defined by a difference of >13.4 points from baseline to post‐assessment. Achieving the RCI and scoring below the cut‐off of eight points on the PAS scale at post‐assessment was defined as a significant remission.

Data were processed and analysed by means of IBM SPSS 29.0 statistical software and the R packages *mitml* (Grund et al., 2023) for the multiple imputation in the LMM and *r2glmm* (Nakagawa & Schielzeth, 2013) for calculating the *R* ^2^. Effects were regarded as being significant at an α‐level of 0.05. In the case of the secondary outcomes, depressive symptoms and agoraphobic avoidance, a Bonferroni‐corrected α‐level of 0.017 was used to correct for multiple testing.

## RESULTS

### Sample characteristics

In total, *N* = 195 participants were included in this comparison study (*n* = 57 app group, *n* = 138 F2F group). Table 1 depicts the demographic characteristics of the participants enrolled. The two groups differed significantly in the amount of comorbid depressive disorders and employment status, which is why these two variables were included as covariates in the LMM analyses.

**TABLE 1:** Demographic sample characteristics at baseline.

	Total (*N* = 195)^a^	App (*n* = 57)	F2F (*n* = 138)	Test for differences^b^
Gender*: % (*n*)				
Female	74.9 (146)	70.2 (40)	76.8 (106)	χ^2^(1) = 0.94, *p* = .366
Male	25.1 (49)	29.8 (17)	23.2 (32)	
Age: M (SD)	35.75 (11.67)	36.35 (14.40)	35.50 (10.39)	*t*(193) = −0.32, *p* = .645
Employment status: % (*n*)				
Not employed	17.9 (35)	36.8 (21)	10.1 (14)	*χ* ^*2*^(1) = 19.52, *p* = <.001
Diagnosis: % (*n*)				
Panic disorder	93.3 (182)	94.7 (54)	92.8 (128)	*χ* ^*2*^(1) = 0.26, *p* = .759
Agoraphobia	91.3 (178)	93.0 (53)	90.6 (125)	*χ* ^*2*^(1) = 0.29, *p* = .782
Comorbidity: % (*n*)				
At least one comorbid disorder	64.5 (159)	61.4 (35)	90.0 (124)	*χ* ^*2*^(1) = 21.69, *p* = <.001
Comorbid depressive disorder	34.9 (68)	45.6 (26)	30.4 (42)	*χ* ^*2*^(1) = 4.09, *p* = .049

Abbreviations: App, App CBT; F2F, Face‐to‐face CBT.

* All participants either identified as male or female; no one identified as diverse.

^a^ Variations in the total *N* caused by missing values.

^b^ Test for differences dependent on the variable being categorical (chi square test) or metric (*t*‐test).

### Dropout

19.3% (*n* = 11) of the app and 14.5% (*n* = 20) of the F2F group dropped out during the course of the respective study. Dropout rates did not significantly differ between the two groups (*χ* ^*2*^(1) = 0.697, *p* = .398).

### Treatment adherence

#### App treatment

There was considerable variation in the extent to which the app was used. More than 85% of the app participants completed all of the psychoeducation lessons (mean adherence score *M* = 85.86, SD = 33.16). Only half of the participants (56.4%) completed the requisite minimum of five exercises in the modules ‘symptom provocation’ and ‘exposure’. 25% did not document any exercise. On average, participants reached an adherence score of 63% (SD = 42.23) in the exercise modules. 70% of participants made at least eight entries in the ‘daily diary’ and the ‘check‐up’ sections, with an average number of 15.20 (SD = 14.93) entries. The mean adherence score for the self‐monitoring modules was 78% (SD = 30.43). The overall adherence score was 75.6% (SD = 27.72), which was met by 34 out of 55 (61.8%) participants who had consented to the processing of their app data.

#### F2F group

The results of the participant and therapist ratings of homework compliance are described in detail in Cammin‐Nowak et al. (2013). Mean overall compliance with homework was high (*M* = 4.99, SD = 0.65), with only 4.7% of all homework not completed at all. Compliance with in situ exposure exercises was lower than compliance with symptom provocation exercises and reflective homework, with 7.55% of in situ exposure exercises left entirely uncompleted. Homework non‐completion was lower in reflective (0.49%) and symptom provocation homework (2.0%). Therapists rated the mean overall quantity of completed homework as lower than assigned (*M* = 1.34, SD = 0.32), whilst the quality was considered satisfactory (*M* = 1.14, SD = 0.49).

### Severity of panic and agoraphobic symptoms

Table 2 shows the descriptive data of all outcomes at baseline and post‐assessment in the app and F2F group (see Appendix C for a graphical representation of changes in all outcomes from baseline‐ to post‐assessment in the two groups). The two groups did not differ significantly in the severity of panic and agoraphobic symptoms (PAS total score) at baseline. Within‐group differences in the PAS total score from baseline to post‐assessment were Cohen's *d* = 1.11 for the app and Cohen's *d* = 1.34 for the F2F group.

**TABLE 2:** Descriptive data of the outcome variables at baseline and post‐assessment in the two groups.

Outcome	Time point	*N* total	*N* app	App M (SD)	N F2F	F2F M (SD)
PAS	Baseline	195	57	27.51 (8.49)	138	27.11 (9.99)
	Post	166	46	19.96 (9.34)^a,^, *, ^b,*^	120	14.68 (8.56)^a,^, *, ^b,*^
BDI	Baseline	195	57	15 (10.23)	138	15.43 (8.00)
	Post	166	46	10.98 (8.73)^a,^, **	120	8.97 (8.95)^a,^, **
MI_acc_	Baseline	175	57	1.95 (1.00)	118	2.20 (0.67)
	Post	156	46	1.86 (1.03)	110	1.58 (0.69)^a,^, **
MI_alone_	Baseline	175	57	2.55 (0.95)^b,^, **	118	2.91 (0.79)^b,^, **
	Post	156	46	2.17 (1.01)^a,^, **	110	2.10 (0.93)^a,^, **

Abbreviations: acc, accompanied and alone subscales; App, App group; BDI, Beck Depression Inventory (BDI‐II, Hautzinger et al., 2006); F2F, Face‐to‐face CBT group; F3, comorbid depressive disorder: yes/no; unemployed: yes/no; α‐level = .05; PAS, Panic and Agoraphobia Scale (Bandelow, 2016); MI, Mobility Inventory (Chambless et al., 1985).

^a^ Within‐group difference, *t*‐test.

^b^ Between‐group difference, *t*‐test.

* Significant difference at α‐level = .05.

** Bonferroni‐corrected α‐level = .01.

Table 3 shows the results from the LMM in the PAS total score in the intent‐to‐treat and the completer datasets. In the intent‐to‐treat as well as the completer data set, the group × time interaction effect became significant for the total PAS score, indicating that the F2F group had significantly greater reductions in PAS symptoms over time compared to the app group. There was a significant effect of time and unemployment, whilst the overall group effect and the presence of a comorbid depressive disorder did not reach significance. Including the interaction term in the model made a significant difference (intent‐to‐treat: *F*(1, 367.472) = 10.187, *p* = .002, *R* ^2^ = 0.32, completer: *χ* ^*2*^(1) = 11.63, *p* = <.001, *R* ^2^ = 0.32). However, the effect of adding the interaction was small (intent‐to‐treat: Diff(*R* ^*2*full^ *– R* ^*2*null^) = 0.010; completer: Diff(*R* ^*2*full^ *– R* ^*2*null^) = 0.011).

**TABLE 3:** Results of the LMM in all outcome measures and the intent‐to‐treat and completer‐data sets.

Outcome	Factor	Data set							
		Intent‐to‐treat				Completer			
		Estimate	SE	*t*‐value	*p*	Estimate	SE	*t*‐value	*p*
PAS	Intercept	20.45	0.83	24.60	<0.001	25.25	1.55	16.33	<0.001
	Group	0.71	0.68	1.06	0.291	1.45	1.64	0.88	0.378
	Time	5.04	0.37	13.65	<0.001	−7.50	1.31	−5.72	<0.001
	F3	2.11	1.22	1.73	0.084	1.08	1.33	0.81	0.418
	Unemployed	4.35	1.58	2.75	0.006	4.49	1.68	2.67	0.008
	Group × Time	−1.26	0.39	−3.19	0.001	−5.34	1.55	−3.45	<0.001
BDI^a^	Intercept	9.69	0.77	12.49	<0.001	10.20	1.37	7.44	<0.001
	Group	−0.16	0.62	−0.26	0.792	2.86	1.45	1.97	0.050
	Time	2.45	0.32	7.74	<0.001	−2.76	1.06	−2.61	0.010
	F3	7.24	1.14	6.35	<0.001	6.75	1.21	5.60	<0.001
	Unemployed	1.37	1.45	0.94	0.347	1.92	1.52	1.26	0.209
	Group × Time	−0.79	0.32	−2.49	0.013	−3.46	1.25	−2.77	0.006
MI_acc_ ^a^	Intercept	1.79	0.08	22.99	<0.001	1.87	0.14	13.33	<0.001
	Group	−0.03	0.06	−0.55	0.586	0.31	0.15	2.07	<0.040
	Time	0.19	0.03	6.95	<0.001	−0.14	0.09	−1.53	0.123
	F3	0.08	0.11	0.73	0.463	0.05	0.13	0.40	0.689
	Unemployed	0.25	0.15	1.70	0.089	0.29	0.16	1.77	0.079
	Group × Time	−0.12	0.03	−4.32	<0.001	−0.51	0.11	−4.64	<0.001
MI_alone_ ^a^	Intercept	2.33	0.09	26.47	<0.001	2.44	0.15	15.42	<0.001
	Group	−0.11	0.07	−1.52	0.128	0.41	0.17	2.43	<0.001
	Time	0.30	0.03	9.67	<0.001	−0.40	0.11	−3.80	0.256
	F3	0.18	0.13	1.34	0.182	0.17	0.15	1.14	0.372
	Unemployed	0.13	0.17	0.76	0.447	0.16	0.18	0.90	0.372
	Group × Time	−0.10	0.03	−3.17	0.002	−0.42	0.13	−3.32	0.001

Abbreviations: acc, accompanied and alone subscales; BDI, Beck Depression Inventory (BDI‐II, Hautzinger et al., 2006); F3, comorbid depressive disorder: yes/no; unemployed: yes/no; α‐level = .05; MI, Mobility Inventory (Chambless et al., 1985); PAS, Panic and Agoraphobia Scale (Bandelow, 2016).

^a^ Bonferroni‐corrected α‐level = .017.

### Response and remission

13% (*n* = 6) of the app participants and 47.1% (*n* = 56) of the F2F participants achieved reliable improvements. The difference between the two groups was significant (*χ* ^*2*^(1) = 16.365, *p* = <0.001). 4.3% (*n* = 2) of the app participants and 12.7% (*n* = 15) of the F2F participants (*n* = 15) met the remission criteria. However, the difference between the two groups was not statistically significant (*χ* ^*2*^(1) = 2.492, *p* = .156).

### Depressive symptoms

The two groups did not differ with regards to depressive symptoms at baseline (Table 2). In the LMM, the group × time interaction gained significance in the intent‐to‐treat and the completer‐data sets (Table 3). It made a significant difference whether the interaction term was included in the model or not (intent‐to‐treat: *F*(1, 513.316) = 6.175, *p* = .013, *R* ^2^ = .24, Completer: *χ* ^*2*^(1) = 7.609, *p* ≤ 0.006, *R* ^2^ = .22). However, adding the interaction to the model had a small effect (intent‐to‐treat: Diff(*R* ^2full^ *– R* ^2null^) = 0.006; completer: Diff(*R* ^2full^ *– R* ^2null^) = 0.007). Hence, the F2F group exhibited a significantly greater reduction in depressive symptoms compared to the app group.

### Agoraphobic avoidance

The two groups showed no significant difference on the MI accompanying scale at baseline (Table 2). The group × time interaction gained significance in the LMM (Table 3). The F2F group showed a significantly greater reduction in agoraphobic avoidance in accompanied situations compared to the app group. The LMM including the interaction term explained significantly more variance in the data than the null model (intent‐to‐treat: *F*(1, 273.102) = 18.675, *p* ≤ 0.001, *R* ^2^ = .12, completer: *χ* ^*2*^(1) = 20.363, *p* ≤ 0.001, *R* ^2^ = .12). Adding the interaction to the model had a small effect (intent‐to‐treat: Diff(*R* ^2full^ *– R* ^2null^) = 0.018; completer: Diff(*R* ^2full^ *– R* ^2null^) = 0.020).

In the case of the unaccompanied MI scale, the two groups differed significantly at baseline (Table 2). The group × time interaction in the LMM was significant (Table 3), indicating a significantly greater reduction in agoraphobic avoidance in unaccompanied situations in the F2F group compared to the app group. It also made a significant difference whether the interaction term was included in the model or not (intent‐to‐treat: *F*(1, 384.401) = 10.076, *p* = .002, *R* ^2^ = .15, completer: *χ* ^*2*^(1) = 10.73, *p* = .001, *R* ^2^ = .15). Again, adding the interaction to the model only had a small effect (intent‐to‐treat: Diff(*R* ^2full^ *– R* ^2null^) = 0.009; completer: Diff(*R* ^2full^ *– R* ^2null^) = 0.010).

## DISCUSSION

### Main findings

Exposure‐based CBT is considered the treatment of first choice for panic disorder and agoraphobia. Unfortunately, only a minority of patients with PD/A receive adequate care, often due to long waiting times or other barriers to psychotherapy. Digital mental health interventions, including smartphone apps as low‐threshold treatment approaches, could help bridge these gaps. Previous studies have already demonstrated the effectiveness of such DMHIs, but primarily in comparison to waitlist control groups or in formats that included limited therapist contact. Therefore, the aim of the current study was to examine whether a smartphone‐based self‐management DMHI for PD/A can match the effectiveness of the ‘gold standard’ CBT for PD/A.

Consistent with prior findings, both the app group and the F2F group led to significant reductions in PD/A symptoms. The within‐group effect sizes from baseline to post‐assessment were similar for the app group (*d* = 1.11) and the F2F group (*d* = 1.34), aligning with effect sizes of DMHIs reported in meta‐analyses (e.g. Domhardt et al., 2020; Pauley et al., 2023; Stech et al., 2020). This is quite remarkable, given that effects in the app group were achieved entirely through self‐help. Nevertheless, F2F CBT resulted in significantly more reliable symptom improvement and clearly outperformed the app‐based intervention in terms of efficacy. F2F CBT was also shown to be superior to the smartphone‐based DMHI in reducing depressive symptoms and agoraphobic avoidance. This is important as previous meta‐analyses suggested that there might be no meaningful differences between guided and unguided DMHIs in anxiety disorders (Pauley et al., 2023) and that therapist‐assisted DMHIs can be equally effective in targeting PD/A symptoms as F2F treatments (Domhardt et al., 2020). These findings may not be generalized to stand‐alone DMHIs as our comparison with F2F CBT shows. It should also be noted that both meta‐analyses aggregated data across heterogeneous populations and diverse interventions, leaving open the critical question of which intervention works best, for whom and under what circumstances in a self‐help DMHI format. Consistent with the findings of Seegan et al. (2023) our analyses suggest that when both are based on the same manual, face‐to‐face therapy is superior to app therapy in panic and agoraphobia patients. We, thus, see a clear need for further research elucidating differential effectiveness and mechanisms of action in DMHIs in different populations.

Interestingly, participants of both the DMHI and F2F intervention showed similar low dropout and high adherence patterns. This is somewhat surprising given that attrition in DMHI studies is usually high (Cavanagh, 2010). Given that individuals who do not complete DMHI treatment programmes suffer relevant adverse effects from dropping out (Mehrmann & Gerlach, 2024), this is an especially encouraging finding, suggesting that both interventions were similarly appealing and tolerable to participants. This supports the view that stand‐alone DMHIs may serve as a supplementary tool or a component of a stepped‐care approach when traditional treatments are not accessible. They may help alleviate anxiety symptoms for individuals in need, though they are unlikely to fully replace F2F CBT. Further research, however, should clarify whether individual differences moderate the tolerability and effectiveness of self‐management DMHIs.

### Strengths and limitations

Our analyses were based on a post hoc comparison of two arms from two separate RCTs. One of the study's principal strengths was the availability of data from both trials, which permitted a statistical analysis that extended well beyond the conventional approach of comparing effect sizes, a methodology more commonly employed in benchmark studies, for instance. Moreover, the trials were highly comparable with regard to inclusion and exclusion criteria, outcome measures and interventions. Furthermore, participants in both groups showed comparable severity levels of PD/A symptoms, and in both cases, more detailed analyses of adherence patterns were available in subsequent studies.

However, this type of study does come with limitations. Our analyses were no direct comparison, but rather a post hoc analysis of disparate data sets. Therefore, results may be affected by uncontrollable biases. Furthermore, group sizes were quite different and, thus, results may have been biased in favour of the bigger F2F group. Also, participants in the F2F group were not allowed to take medication, whereas those in the app group were allowed to take medication. This may have distorted the comparisons between the two samples. In addition, although the diagnostic criteria for panic disorder with agoraphobia are largely comparable between DSM‐IV‐TR and DSM‐5, differences in the clinical interview methods employed may have impacted the comparability of the patient populations. Taken together, these methodological differences may limit group comparability and reduce the ability to draw causal inferences.

Furthermore, adherence was operationalized differently across the two studies, which may constrain direct comparability. Whilst a 75% adherence threshold is sometimes used as an indicator of compliance, this operational definition is not universally standardized. Such inconsistency could introduce bias or uncertainty when interpreting differences in adherence between conditions.

The generalizability of our findings regarding the self‐management app may also be limited by the fact that all participants received a structured clinical interview prior to app use. Research suggests that conducting a diagnostic interview before offering a stand‐alone intervention may influence intervention outcomes, particularly when compared to interventions with no contact (Johansson & Andersson, 2012). Moreover, the repeated assessments may limit the transferability of adherence and dropout rates to routine care.

Finally, the two data sets were selected because both were based on the same CBT treatment manual. Nevertheless, our findings may not generalize to other CBT approaches, and a comparison based on different data sets might have yielded different results.

Future studies should replicate and expand upon the current study's findings by comparing the smartphone‐based self‐management tool with F2F CBT in a controlled trial with sufficient power and, for instance, including analyses on long‐term effects. Analyses of underlying working mechanisms and adherence behaviour is of great interest, too. Such studies could be designed to take advantage of inherent opportunities to monitor behavioural data from smartphone‐based applications, such as GPS data during exposure (White et al., 2014). It would also be of great interest to examine whether the use of the app ‘Mindable’ in blended care and, thus, therapist assistance is as effective as F2F CBT.

## CONCLUSIONS

A self‐management smartphone app can effectively reduce panic and agoraphobic symptoms, depressive symptoms and agoraphobic avoidance. However, its effectiveness remains inferior to F2F CBT and is, thus, no substitute for interventions with therapeutic contact. Even if the similar dropout and adherence data of this specific study suggest that self‐management DMHIs and F2F interventions are similarly tolerable and can motivate people to engage in interventions, it is important to counteract the impression that smartphone‐based self‐management DMHIs are equivalent to F2F CBT. Smartphone‐based self‐management DMHIs should, thus, be implemented if CBT with therapeutic contact is not available or wanted, for instance, to bridge waiting time for psychotherapy or if implemented in stepped care. Further research is required to provide additional evidence and a deeper understanding of the efficacy of smartphone‐based self‐management DMHIs, and more therapy places for people suffering from PD/A should be made available in a timely manner.

## AUTHOR CONTRIBUTIONS

**Justine Spies:** Data curation; formal analysis; investigation; visualization; writing – original draft; writing – review and editing. **Thomas Lang:** Conceptualization; formal analysis; funding acquisition; project administration; resources; supervision; validation; visualization. **Alexander L. Gerlach:** Writing – review and editing. **Tilo Kircher:** Writing – review and editing. **Alfons Hamm:** Writing – review and editing. **Georg W. Alpers:** Writing – review and editing. **Thomas Fydrich:** Writing – review and editing. **Volker Arolt:** Writing – review and editing. **Jürgen Deckert:** Writing – review and editing. **Andreas Ströhle:** Writing – review and editing. **Hans‐Ulrich Wittchen:** Writing – review and editing. **Sylvia Helbig‐Lang:** Conceptualization; formal analysis; funding acquisition; methodology; project administration; resources; supervision; validation; visualization; writing – original draft; writing – review and editing.

## FUNDING INFORMATION

This research was funded by the Christoph Dornier Stiftung and by the Mindable Health GmbH.

## CONFLICT OF INTEREST STATEMENT

The study was funded by Mindable GmbH and the Christoph Dornier Stiftung and conducted within an accreditation of the Federal Institute for Drugs and Medical Devices (BfArM).

## ETHICS STATEMENT

DHA: Ethical approval for the study was granted by the ethical committee of the German Society for Psychology (reference number: LangThomas2020‐12‐14VA).

F2F: The RTC project was approved by the Ethics Committee of the Medical Faculty of the Technical University of Dresden (EK 164082006).

## INFORMED CONSENT

Informed consent was obtained from all participants included in the study.

## TRIAL REGISTRATION

DHA: Registration number: DRKS00029090, registered on 10.06.2022.

F2F: The study was registered with the ISRCTN: ISRCTN80046034.

## Figures and Tables

**FIGURE C1:**
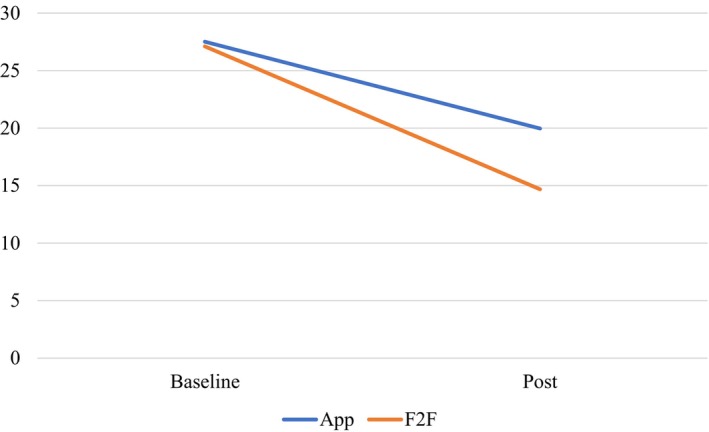
Baseline to post‐assessment changes in the PAS scores in the app and F2F group.

**FIGURE C2:**
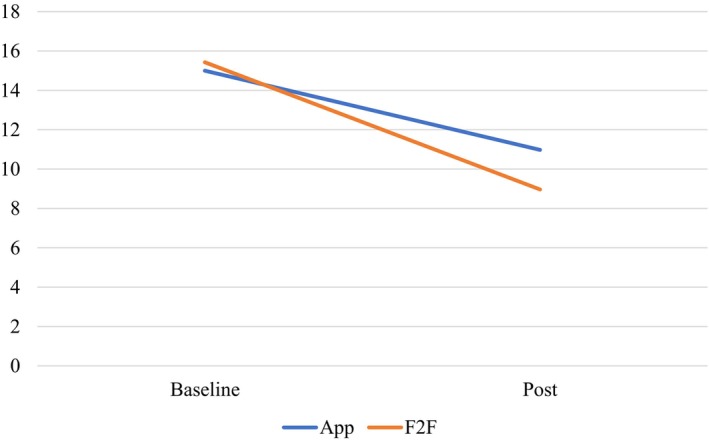
Baseline to post‐assessment changes in the BDI‐II scores in the app and F2F group.

**FIGURE C3:**
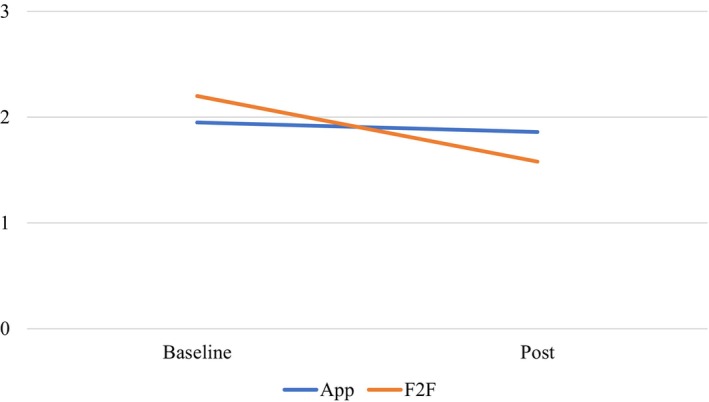
Baseline to post‐assessment changes in the MI accompanied scores in the app and F2F group.

**FIGURE C4:**
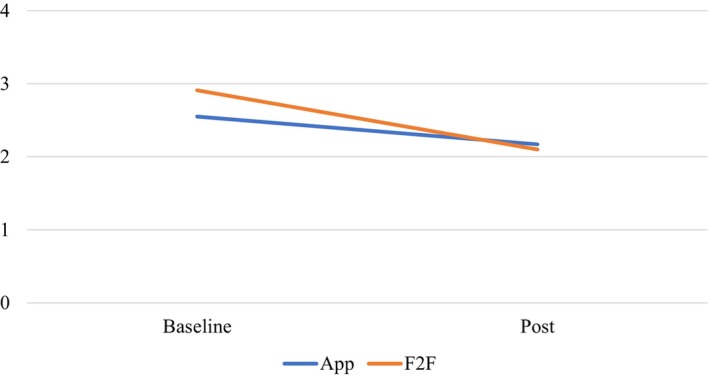
Baseline to post‐assessment changes in the MI alone scores in the app and F2F group.

## Data Availability

The data that support the findings of this study are available from the corresponding author upon reasonable request.

## APPENDIX D Comparative table of core intervention elements

**Table:**

F2F treatment	App adaptation
Psychoeducation (Session 1–3) Vicious cycle of anxiety What is anxiety? Explanations on panic attacks, panic disorder and agoraphobia Negative effects of safety and avoidance behaviours	Same content prepared for self‐study; recommendation to start with this app content
Symptom provocation (Session 4–5) Symptom hierarchy Conducting exercises (such as shaking one's head and head between the knees)	Provided instructions and recordings for the same exercises
Exposure in vivo 1 (Session 6–8) Anxiety hierarchy Preparation of homework exposure exercises in three standard situations: bus, department store, forest Repeating each exercise three times between sessions	Suggestions for standardized exercises, including planning and recording of exposure exercises Participants were free to choose suitable exercises
Anticipatory anxiety (Session 9) What have I learned so far? How does anticipatory anxiety change?	No equivalent app content, anticipatory anxiety could be monitored in the diary function of the app
Exposure in vivo 2 (Session 10–12) Guidance for self‐exposure in vivo in ideographically salient situations	Option of creating, planning and recording own exercises
Booster sessions (Session 13–14) Discussion of individual exposure during booster period; relapse prevention	No equivalent app content

Abbreviation: F2F, Face‐to‐face.
